# Biological Activities of Cationicity-Enhanced and Hydrophobicity-Optimized Analogues of an Antimicrobial Peptide, Dermaseptin-PS3, from the Skin Secretion of *Phyllomedusa sauvagii*

**DOI:** 10.3390/toxins10080320

**Published:** 2018-08-07

**Authors:** Yining Tan, Xiaoling Chen, Chengbang Ma, Xinping Xi, Lei Wang, Mei Zhou, James F. Burrows, Hang Fai Kwok, Tianbao Chen

**Affiliations:** 1 Natural Drug Discovery Group, School of Pharmacy, Queen’s University Belfast, Belfast BT9 7BL, Northern Ireland, UK; ytan07@qub.ac.uk (Y.T.); c.ma@qub.ac.uk (C.M.); l.wang@qub.ac.uk (L.W.); m.zhou@qub.ac.uk (M.Z.); j.burrows@qub.ac.uk (J.F.B.); 2 Faculty of Health Sciences, University of Macau, Avenida de Universidade, Taipa, Macau, China; hfkwok@umac.mo

**Keywords:** antimicrobial peptide (AMP), dermaseptin, anuran skin secretion, drug design, antimicrobial activity, anticancer activity

## Abstract

The skin secretions of the subfamily Phyllomedusinae have long been known to contain a number of compounds with antimicrobial potential. Herein, a biosynthetic dermaseptin-precursor cDNA was obtained from a *Phyllomedusa sauvagii* skin secretion-derived cDNA library, and thereafter, the presence of the mature peptide, namely dermaseptin-PS3 (DPS3), was confirmed by LC–MS/MS. Moreover, this naturally occurring peptide was utilized to design two analogues, K^5, 17^-DPS3 (introducing two lysine residues at positions 5 and 17 to replace acidic amino acids) and L^10, 11^-DPS3 (replacing two neutral amino acids with the hydrophobic amino acid, leucine), improving its cationicity on the polar/unipolar face and hydrophobicity in a highly conserved sequence motif, respectively. The results in regard to the two analogues show that either increasing cationicity, or hydrophobicity, enhance the antimicrobial activity. Also, the latter analogue had an enhanced anticancer activity, with pretreatment of H157 cells with 1 µM L^10, 11^-DPS3 decreasing viability by approximately 78%, even though this concentration of peptide exhibited no haemolytic effect. However, it must be noted that in comparison to the initial peptide, both analogues demonstrate higher membrane-rupturing capacity towards mammalian red blood cells.

## 1. Introduction

Of the many anuran skin-derived peptides that are known, dermaseptin and dermaseptin-like peptides are the most remarkable candidates for developing new antibiotics in Hylidae frogs [1,2,3]. Although there is much heterogeneity in either the peptide sequence, or length, among dermaseptins, the family nevertheless shares several common structural characteristics, including Trp at position 3 and a conserved sequence of AA(G)KAALG(N)A in the mid-region [4]. In addition, dermaseptins commonly possess a high propensity to adopt an α-helical conformation in hydrophobic media, since the first dermaseptin peptide with 80% of α-helical conformation was isolated from Hylidae frogs [3,5,6]. Pharmacologically, apart from broad-spectrum antimicrobial activity (e.g., dermaseptin S4 and B), haemolytic activity and anticancer activity have been reported (dermaseptin-PH and B2) [3,7,8]. 

Numerous studies indicate that the net charge is a key factor influencing the binding of AMPs to membranes, as AMPs bind to the membrane by electrostatic interaction and competitively replace the divalent cation [9,10,11]. Therefore, it is believed that changing the number of positive charges present in an AMP can likely change its membrane binding ability, resulting in a change in antimicrobial activity. Also, in general, approximately half of AMP amino acid residues are hydrophobic, and their hydrophobicity and their activity can be altered by changing the number of Leu, Ile and Val residues in these peptides. However, both antibacterial and haemolytic activities of these AMPs tend to get increased simultaneously by increasing the hydrophobicity, due to the fact that the hydrophobic groups play a key role in their insertion into the cell membrane [10].

Herein, we describe the discovery of a biosynthetic precursor, preprodermaseptin, encoding an antimicrobial peptide, DPS3, from the skin secretion of *Phyllomedusa sauvagii* using a combination of shotgun cloning and mass spectrometry. The corresponding chemically synthesised replicate exerted weak antibacterial activity towards pathogenic microorganisms and weak cytotoxic activity towards tumour cells. Therefore, we designed two analogues of this naturally occurring peptide, K^5, 17^-DPS3 and L^10, 11^-DPS3, to potentially optimize its cationicity on the polar/unipolar face and hydrophobicity in conserved sequence motif of dermaseptin, respectively. 

## 2. Results

### 2.1. Molecular Cloning of a DPS1 Precursor cDNA from a Skin Secretion-Derived cDNA Library

**Figure 1:**
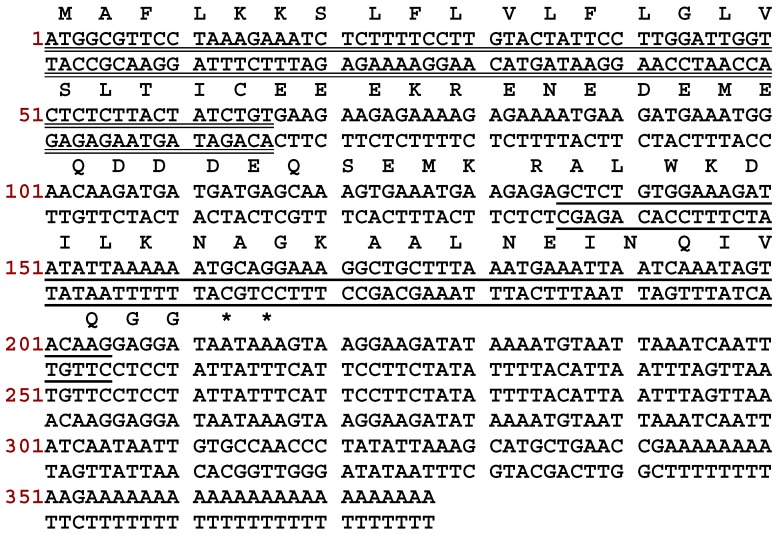
Nucleotide and translated open reading frame amino acid sequence of the cDNA encoding the biosynthetic precursor of a novel peptide from the skin secretion of *Phyllomedusa sauvagii*. The putative N-terminal signal peptide sequence is double-underscored, putative mature peptide sequence is single-underscored and an asterisk indicates the stop codon.

Using the shotgun cloning strategy, the nucleotide sequence of a full-length biosynthetic precursor-encoding cDNA was consistently cloned among the artificially reconstructed cutaneous secretion-derived cDNA library from *Phyllomedusa sauvagii*. More specifically, the domain architecture of this preprodermaseptin transcript (Figure 1) compromises 70 amino acid residues, encoding a single copy of a peptide termed DPS3, where the C-terminus was subjected to post-translational modification with carboxyl-terminal amide formation. From the translated open reading frame, the KR is a typical convertase processing site in vivo and the resulting mature peptide consisted of 23 amino acid residues (ALWKDILKNAGKAALNEINQIVQ-amide). The cDNA precursor was deposited in GenBank database under an accession no. of MH536746. 

### 2.2. Isolation and Structural Characterisation of DPS3

**Figure 2:**
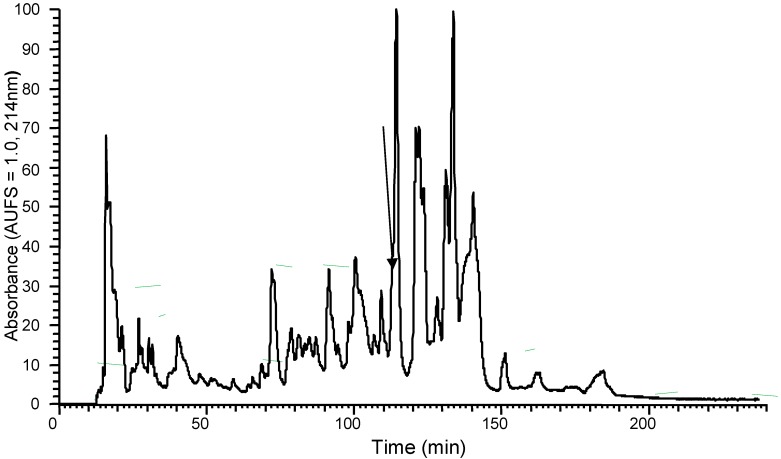
Region of RP–HPLC chromatogram of *Phyllomedusa sauvagii* skin secretion indicating the absorbance peak by an arrow that corresponds to DPS3.

The predicted amino acid sequence identified via cDNA cloning suggested the existence of a peptide in the skin secretion of *Phyllomedusa sauvagii*, so the lyophilized skin secretion was directly analysed to determine if this peptide was present. The presence of the mature DPS3 peptide was confirmed by RP–HPLC isolation, with the retention time at approximately 108 min, and MS/MS fragmentation sequencing (Figure S1, Figure 2 and Table 1, respectively). 

### 2.3. Physicochemical Properties and Secondary Structures of DPS3 and Its Analogues

**Figure 3:**
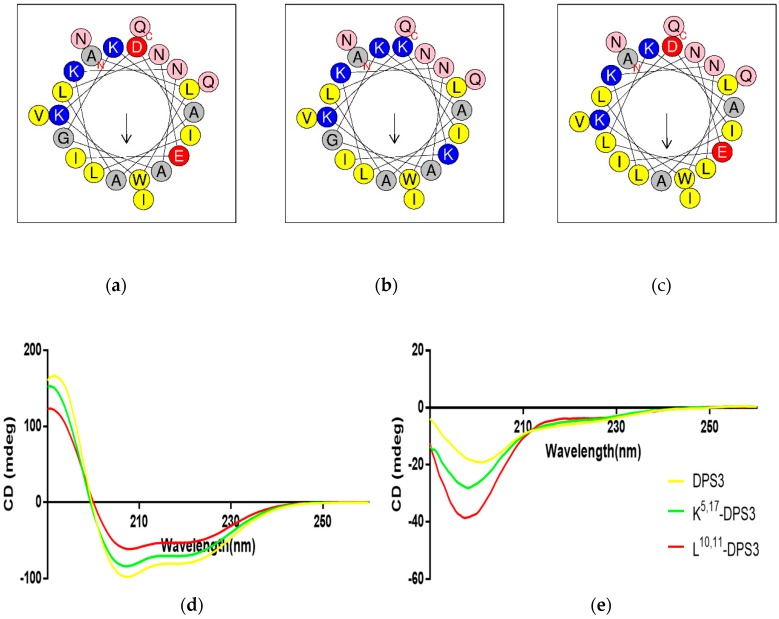
Predicted helical wheel projections of the three peptides, (**a**) DPS3, (**b**) K^5,17^-DPS3 and (**c**) L^10, 11^-DPS3; CDspectra recorded for 100 μM of DPS3 (yellow), K^5, 17^-DPS3 (green) and L^10, 11^-DPS3 (red) peptides in (**d**) 10 mM NH_4_Ac/water solution and in (**e**) 50% TFE/10 mM NH_4_Ac/water solution.

Both DPS3 and L^10, 11^-DPS3 possessed the same net positive charge of +2, which increased to +6 in the case of the cationicity-enhanced analogue (Table 2). Additionally, DPS3 and K^5, 17^-DPS3 had a similar degree of hydrophobicity, which was increased in L^10, 11^-DPS3. The helical wheel projects showed that DPS3 and its analogues have the same direction of summed vectors of hydrophobicity (Figure 3). Meanwhile, K^5, 17^-DPS3 had one more positive charge on both hydrophilic and hydrophobic faces than the other two analogues, and L^10, 11^-DPS3 showed an enlarged hydrophobic face. Also, although these three peptides existed in random coils in aqueous solution, they all adopted α-helical conformations in membrane-mimicking solution, presenting obviously negative peaks at 222 nm and 208 nm, with the natural peptide presenting the largest proportion of α-helical domain (44.9% of its secondary structure) (Figure 3 and Table 2). 

### 2.4. Antimicrobial Activity

The parent peptide, DPS3, generally showed weak antimicrobial activity, although it did exhibit better activity against Gram-negative bacteria. As expected, when compared to the parent peptide, both artificial analogues displayed enhanced antimicrobial activity against all the microorganisms examined (Table 3)**.** In particular, K^5, 17^-DPS3 displayed MIC values of 8 µM or less against Gram-positive (*Staphylococcus aureus*) and Gram-negative (*Escherichia coli*) bacteria, as well as yeast (*Candida albicans*). 

### 2.5. Cytotoxicity of Peptides on Human Cancer and Normal Cells 

**Figure 4:**
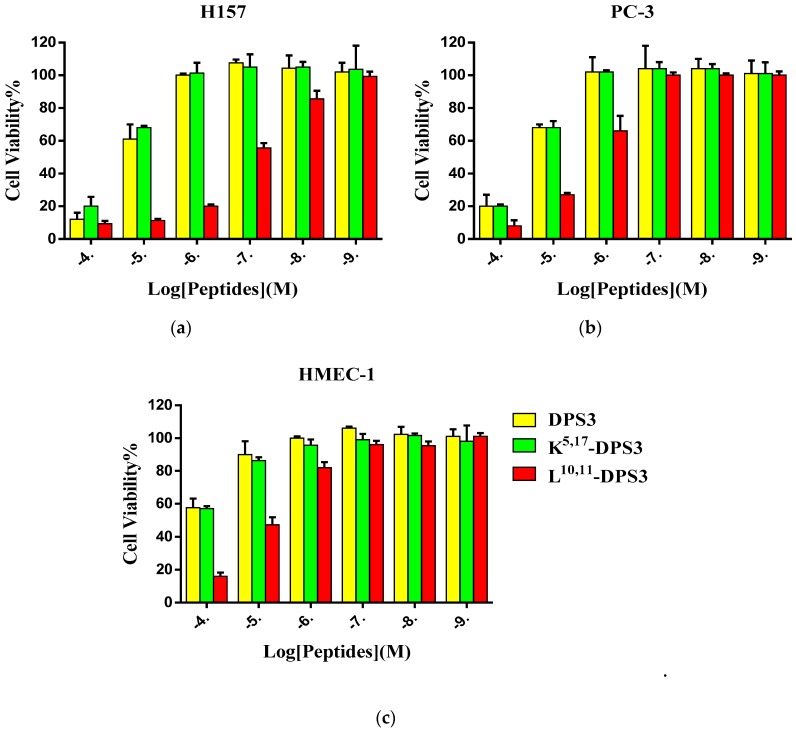
The cytotoxic effect of DPS3 (yellow), K^5, 17^-DPS3 (green) and L^10, 11^-DPS3 (red) on the human cancer cell lines (**a**) H157 and (**b**) PC-3, and normal cell line HMEC-1 (**c**).

DPS3 and its two artificial analogues all exhibited inhibitory effects on the proliferation of the two tested human cancer cell lines and normal cell line (Figure 4). Increasing the cationicity of the parent peptide had no significant influence on its antiproliferative activity, whereas altering its hydrophobicity markedly enhanced its antiproliferative activity, with this peptide exhibiting an IC_50_ value more than 10-fold lower than either of the other peptides (Table 4). 

### 2.6. Haemolysis Activity 

**Figure 5:**
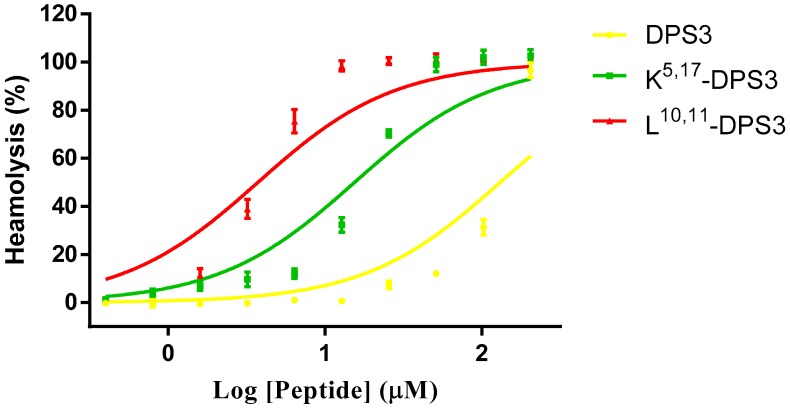
Haemolytic activity of DPS3 (yellow), K^5, 17^-DPS3 (green) and L^10, 11^-DPS3 (red) against horse red blood cells. The HC_50s_ of DPS3, K^5, 17^-DPS3 and L^10, 11^-DPS3 are 138.1, 14.98 and 3.44, respectively.

All three peptides exhibited some haemolytic activity against healthy red blood cells (Figure 5). However, both artificial analogues exhibited a greater effect than the parent peptide, with the L^10, 11^-DPS3 analogue showing marked haemolysis even at lower concentrations. The HC_50s_ of DPS3, K^5, 17^-DPS3 and L^10, 11^-DPS3 are 138.1, 14.98 and 3.44, respectively.

## 3. Discussion

Typically, most naturally occurring dermaseptins are 28 to 34 amino acid residues in length [12,13,14], therefore, the 23-mer DPS3 reported here is relatively short for this family. Huang and colleagues have previously isolated a similar-length dermaseptin, dermaseptin-PH [3]. Compared with other similar dermaseptins, the antimicrobial activity of DPS3 and dermaseptin-PH indicate that these two native truncated dermaseptins are less potent as AMPs than other longer dermaseptin peptides, possibly suggesting increasing peptide length in this family is potentially related to a higher antimicrobial activity. Besides, the physicochemical properties, including charge and hydrophobicity, are the main factors affecting antimicrobial activity, and therefore they are considered as one of the design parameters to optimize in AMPs [7,15,16,17]. In terms of the antibacterial mechanism of action of AMPs, it is mostly thought to concern the electrostatic interaction and hydrophobic engagement between AMPs and bacterial cell membranes [18]. Compared with other members of the dermaseptin family (Figure S2), DPS3 shares sequence similarities and they are canonical cationic and α-helical amphipathic peptides [1,3,5,8,19,20]. Besides, the conformational transition feature from coil to helix upon binding to lipid bilayers, in general, concerns a membrane-damaging mode of action in the dermaseptin family. In particular, the polycationic properties, as well as a large number of hydrophobic amino acids in the primary structure, and conformational alternation from random coil to helical frame among dermaseptins, along with their membrane-lytic activity, suggest their mechanism of action is likely to involve membrane disruption. Indeed, previous studies have found leakage and morphological alterations in the artificial bacterial membrane after treatment with fluorescent-labelled dermaseptins [21,22]. More recently, using the electron microscopy, a dermaseptin peptide, DS1, was found to distort the cell wall surface, proposing that the cytolysis or cell membrane disruption of *C. albicans* eventually cause cell death [23]. 

Early research has revealed a strong correlation between α-helical domain and antimicrobial activity, involving the local fusion of the membrane leaflets, pore formation, cracks, as well as the depth of membrane insertion [24,25,26]. Also, the α-helical conformation of dermaseptins is normally considered as one of the main factors in the hydrophobic interaction of AMPs and lipid layer [24,26]. In our investigation, the CD spectra of the three peptides showed that the amount of helical conformation is similar across all three. Our results indicate that an increase in antimicrobial activity can be achieved via optimization of cationic or hydrophobic properties of the dermaseptin peptides through residue substitution. However, both artificial analogues induced more haemolysis than the parent peptide, though K^5, 17^-DPS3 showed minimal haemolysis at the concentrations that exhibited antimicrobial activity, indicating it could still represent an interesting AMP. It is often supposed that the electrostatic interaction between anionic molecules (such as LPS, teichoic acids or acidic phospholipids) and positively charged AMPs is an essential step allowing the cationic peptides to selectively aggregate on the bacterial membrane; therefore, it is also believed that changing the number of positive charges present in an AMP can likely change its membrane binding ability [9,10,11]. Also, the selective interactions between lysine/arginine residues and anionic lipid membranes and reduced selectivity upon increasing hydrophobic properties of antimicrobials have been supported by X-ray scattering data [27,28]. We speculate that the cationicity-enhanced analogue shows more sensitive membrane binding ability towards bacterial cells than mammalian cells, therefore resulting in less haemolysis. The electrical attraction of these peptides to the membrane is important for their antimicrobial activity, and this could explain why the cationicity-enhanced analogue shows more potent antimicrobial activity than L^10, 11^-DPS3, but less haemolysis. Similarly, K_4_K_20_-S4, a dual lysine-substituted dermaseptin-S4, also shown increased antimicrobial activity, along with two-fold haemolytic potency and higher lipophilic affinity [29]. Previously, 2D-NMR has shown that the consensus motif AA(G)KAALG(N) among dermaseptins is adopting a well-defined α-helical structure [1,7]. Herein, we enhanced the hydrophobicity in this highly conserved motif with amphipathic α-helix and found more potent cytolytic action towards mammalian erythrocytes. On the other hand, as the hydrophobicity is important for membrane disruption, increasing the hydrophobicity could improve the peptides’ ability to disrupt membranes, but not in a way that favours microorganism selectivity. In this regard, we proposed that it possibly results from stronger hydrophobic interaction within the core of the bacterial membrane. Taken together, the data presented indicate that it is possible to improve the membrane-lytic activity of these AMPs without increasing the peptide length. 

DPS3, and its analogues, all exhibit antiproliferative effects on the tested cancer cells, although L^10, 11^-DPS3 exhibited an enhanced antiproliferative impact. Recent studies have shown that cancer cell membranes, similar to the bacterial membrane, carry a negative charge due to overexpression of anionic molecules, such as phosphatidylserine, sialic acid and glycosaminoglycans (GAGs) [7,17,18]. Therefore, although it is unclear how the dermaseptins mediate their anticancer activity, it could result from their interaction with and disruption of the cell membrane, similar to their antimicrobial action. However, dermaseptins have been found to interact with, and aggregate on, the surface of cancer cells, as well as being able to penetrate into cancer cells, without compromising the cell membrane [8,14,19,22]. Also, Dos Santos and colleagues found that an Alexa- and biotin-labelled version of the dermaseptin peptide, DRS-B2, could internalise into cancer cells, and implicated its non-protein binding partner GAGs, suggesting GAGs are possibly involved in dermaseptin internalization [8]. Notably, the viability of H157 cells pretreated with L^10, 11^-DPS3 (1 µM) decreased by approximately 78%, whilst no membrane lysis was observed in mammalian erythrocytes exposed to the same concentration of peptide, possibly suggesting that a nonlytic mechanism may be involved, at least at lower concentrations. At higher peptide concentrations (>1 µM), the higher anticancer cell impact is consistent with increasing haemolysis activity, suggesting that this is possibly due to cell membrane disruption. However, further investigations will be needed to confirm this further.

## 4. Conclusions

The 23-mer peptide DPS3 reported here is relatively short in comparison to other naturally occurring dermaseptins, which are commonly 28 to 34 amino acids. Although DPS3 exhibited relatively weak bioactivity for dermaseptin family members, we rationally designed two DPS3 analogues with the aim of improving its bioactivity. In particular, K^5, 17^-DPS3 and L^10, 11^-DPS3 both exhibited more potent antimicrobial activity, whilst L^10, 11^-DPS3 also exhibited enhanced anticancer activity, even at concentrations that had minimal impact upon healthy mammalian cells. This would suggest that K^5, 17^-DPS3 and L^10, 11^-DPS3 may have promise as antimicrobial agents, and L^10, 11^-DPS3 could have potential as an anticancer agent. However, further investigations will be required to determine the exact mode of action that these peptides utilise against the microbial and cancer cells.

## 5. Materials and Methods

### 5.1. Acquisition of Phyllomedusa sauvagii Dermal Secretions

Three specimens of *Phyllomedusa sauvagii* (4–6 cm snout-to-vent length) obtained from a commercial source in the United States were exposed to 12 h of light at 20–25 °C daily, and multivitamin-loaded crickets were provided as the fodder three-times/week. Following four-month breeding, dermal secretions were collected via surface electrical stimulation [30]. In summary, the skin surface was moistened with deionized water, followed by mild transdermal electric stimulation (5 V, 100 Hz, 140 ms pulse width). Finally, the secretions were collected by gently flushing the frog skin with deionized water, and they were lyophilized and stored at −20 °C until mRNA extraction. Animal procedure was performed according to the guidelines in the UK Animal (Scientific, Procedures) Act 1986, project license PPL 2694, issued by the Department of Health, Social Services and Public Safety, Northern Ireland. Procedures had been vetted by the IACUC of Queen’s University Belfast, and approved on 1 March 2011.

### 5.2. Shotgun Cloning of a cDNA Encoding DPS3 Peptide Biosynthetic Precursor

The shotgun cloning employed the rapid amplification of cDNA ends (RACE) technique with a degenerate primer, which has been described previously [31]. Briefly, the mRNA from the skin secretion of *Phyllomedusa camba* was reverse-transcript to the cDNA library. The 3′-RACE was conducted using the cDNA library and the primer (5′-ACTTTCYGAWTTRYAAGMCCAAABATG-3′) that was designed to a segment of the 5′-untranslated region of cDNAs from *Phyllomedusa* species (accession nos. AJ251876, AJ005443). The RACE products were subjected to purification and cloned, and finally sequenced by the ABI 3100 Automatic Capillary Sequencer.

### 5.3. Identification and Analysis of Amino Acid Sequence

The identification of DPS3 from the skin secretion was performed according to our previous study [31]. Briefly, 5 mg of lyophilized skin secretion was separated using the RP–HPLC column (Jupiter C-18 250 mm × 10 mm, Phenomenex, Macclesfield, UK). The fraction was collected every minute and then analyzed using MALDI–TOF MS (Perseptive Biosystems, Voyager DE, Perseptive Biosystems, Framingham, MA, USA) and LCQ-Fleet ion-trap MS for sequencing (Thermo Fisher Scientific, San Francisco, CA, USA).

### 5.4. Design and Synthesis of DPS3 and Its Two Analogues

To investigate the effect of the increasing cationicity and hydrophobicity, the peptide DPS3 was used as the framework to design two analogues where the two acidic amino acids (at positions 5 and 17) and two neutral amino acid residues (at positions 10 and 11) were substituted with lysine and leucine residues, respectively. Accordingly, theses analogues are named K^5, 17^-DPS3 (ALWKKILKNAGKAALNKINQIVQ-NH_2_) and L^10, 11^-DPS3 (ALWKDILKNLLKAALNEINQIVQ-NH_2_). The mean hydrophobicity, hydrophobic moment and helical wheel projections of peptides were predicted by Heliquest (http://heliquest.ipmc.cnrs.fr/). Peptide synthesis was carried out using Tribute peptide synthesizer (Protein Technologies, Tucson, AZ, USA) along with Rink amide resin and standard Fmoc chemistry, which was described in a previous study [32]. 

### 5.5. CD Analysis of Synthetic Peptides

The secondary structure was determined by a CD spectrometer (Jasco J851, Tokyo, Japan). The analysis method was performed as previous study [32]. The obtained spectra were analysed by the BeStSel CD online analysis program (http://bestsel.elte.hu [33]) to calculate the proportion of α-helical conformation of each peptide. 

### 5.6. Antimicrobial Assays

The antimicrobial activity of three peptides was evaluated via the minimal inhibitory concentrations (MICs) against *S. aureus* (NCTC 10788), *E. coli* (NCTC 10418) and *C. albicans* (NCYC 1467) [32]. Microorganisms were inoculated with peptide (1 to 512 μM) in a 96-well plate and determined at 550 nm using a Synergy HT plate reader (Biotech, Winooski, VT, USA). 

### 5.7. Cell Viability of Human Cancer and Normal Cells 

Cell viabilities were achieved using a typical MTT assay [32]. Briefly, non-small cell lung cancer cell line, H157, human prostate carcinoma cell line, PC-3, and dermal microvascular endothelium cell line, HMEC-1, were treated with synthesized peptides from 10^−4^ to 10^−9^ M. After adding MTT, the formazan crystals were dissolved and read by the plate reader at 570 nm.

### 5.8. Haemolysis Assay

The haemolytic activity of each peptide was determined using a 2% suspension of horse blood cells (supplied by TCS Biosciences Ltd., Buckingham, UK) as previous study [32]. The red blood cell suspension was treated by the peptide (512−1 μM) at 37 °C for 2 h. The release of haemoglobin detected by the plate reader at λ550 nm. 1% Triton X-100 and PBS were applied as the positive and negative controls, respectively.

## Figures and Tables

**Table 1:** Predicted b-ion and y-ion MS/MS fragment ion series (singly and doubly charged) of DPS3. The observed ions are indicated by single-underlined. The unit for MS data is *m*/*z*.

#1	b (1+)	b (2+)	Seq.	y (1+)	y (2+)	#2
1	72.04440	36.52584	A			23
2	185.12847	93.06787	L	2478.41923	1239.71325	22
3	371.20779	186.10753	W	2365.33516	1183.17122	21
4	499.30276	250.15502	K	2179.25584	1090.13156	20
5	614.32971	307.66849	D	2051.16087	1026.08407	19
6	727.41378	364.21053	I	1936.13392	968.57060	18
7	840.49785	420.75256	L	1823.04985	912.02856	17
8	968.59282	484.80005	K	1709.96578	855.48653	16
9	1082.63575	541.82151	N	1581.87081	791.43904	15
10	1153.67287	577.34007	A	1467.82788	734.41758	14
11	1210.69434	605.85081	G	1396.79076	698.89902	13
12	1338.78931	669.89829	K	1339.76929	670.38828	12
13	1409.82643	705.41685	A	1211.67432	606.34080	11
14	1480.86355	740.93541	A	1140.63720	570.82224	10
15	1593.94762	797.47745	L	1069.60008	535.30368	9
16	1707.99055	854.49891	N	956.51601	478.76164	8
17	1837.03315	919.02021	E	842.47308	421.74018	7
18	1950.11722	975.56225	I	713.43048	357.21888	6
19	2064.16015	1032.58371	N	600.34641	300.67684	5
20	2192.21873	1096.61300	Q	486.30348	243.65538	4
21	2305.30280	1153.15504	I	358.24490	179.62609	3
22	2404.37122	1202.68925	V	245.16083	123.08405	2
23			Q-Amidated	146.09241	73.54984	1

**Table 2:** Physicochemical properties of DPS3 and its two analogues.

Peptide	Hydrophobicity (H)	Hydrophobic Moment (µH)	% Helix ^1^	Net Charge
ALWKDILKNAGKAALNEINQIVQ-NH_2_	0.373	0.437	44.9	+2
ALWKKILKNAGKAALNKINQIVQ-NH_2_	0.349	0.437	39	+6
ALWKDILKNLLKAALNEINQIVQ-NH_2_	0.508	0.517	28.8	+2

^1^ In 50% 2,2,2-trifluoroethanol (TFE)/10 mM ammonium acetate (NH_4_Ac) solution.

**Table 3:** Antimicrobial activity of the parent DPS3 peptide and its two analogues against various microorganisms.

Microorganisms	DPS3	K^5, 17^-DPS3	L^10, 11^-DPS3
	MIC (µM)		
*S. aureus* (NCTC 10788)	256	8	8
*E. coli* (NCTC 10418)	32	8	16
*C. albicans* (NCYC 1467)	64	4	16

**Table 4:** Induced cytotoxicity of DPS3 and analogues on the human cancer cells. IC_50_s were calculated from the normalized curves in Figure 4 using GraphPad Prism 6 (GraphPad Software, USA).

Peptide	IC_50_ for H157 (µM)	IC_50_ for PC3 (µM)	IC_50_ for HMEC-1 (µM)
DPS3	15.67	18.20	132.10
K^5, 17^-DPS3	18.20	18.20	123.00
L^10, 11^-DPS3	0.12	1.85	8.76

## Supplementary Materials

The following are available online at http://www.mdpi.com/2072-6651/10/8/320/s1, Figure S1: Annotated fragment ion spectrum of DPS3, Figure S2: Alignment of amino acid sequences of DPS3 and other dermaseptins.
