# Supplementary material for: Biological Activities of Cationicity-Enhanced and Hydrophobicity-Optimized Analogues of an Antimicrobial Peptide, Dermaseptin-PS3, from the Skin Secretion of Phyllomedusa sauvagii
Source: Toxins (Basel). 2018 Aug 7;10(8):320. doi: 10.3390/toxins10080320 (PMC6115755; doi:10.3390/toxins10080320)
Supplement: Supplementary file 1 [file toxins-10-00320-s001.pdf]

# Supplementary Materials: Biological Activities of Cationicity-Enhanced and Hydrophobicity-Optimized Analogues of an Antimicrobial Peptide, Dermaseptin-PS3, from the Skin Secretion of *Phyllomedusa sauvagii*

**Yining Tan, Xiaoling Chen, Chengbang Ma, Xinping Xi, Lei Wang, Mei Zhou, James F. Burrows, Hang Fai Kwok and Tianbao Chen**

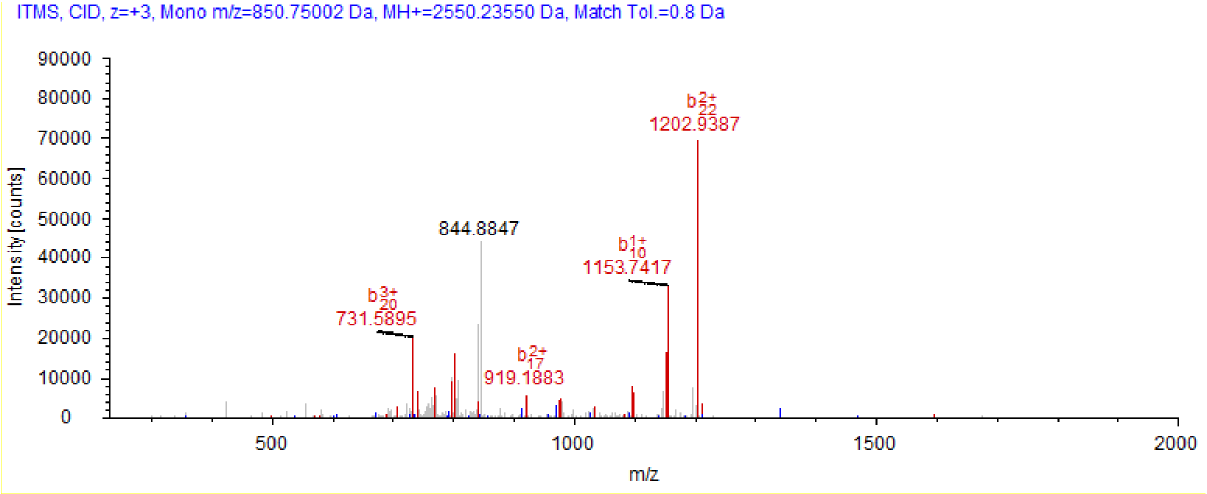


**Figure S1.** Annotated fragment ion spectrum of DPS3.

```
Name                      Sequence                                     Reference

DRS-LIKE            ALW-------KDVLKKIGTVALHAGKAALGAVADTISQ---           [5]
DPS1                ALW-------KTMLKKLGTMALHAGKAALGAAADTISQGTQ            a
DRS-S2              ALW-------FTMLKKLGTMALHAGKAALGAAANTISQGTQ           [19]
DRS-B2              GLWSKIKEVGKEAAKAAAKA---AGKAALGAVSEAV-----           [8]
DPS2                ALW-------KTLLKNVGKA---AGKAVLNAVTDMVNQGEQ           [20]
Dermaseptin-PH      ALW-------KEVLKN-------AGKAALNEINNLVQGGQ-           [3]
DRS-B6              ALW-------KDILKN-------AGKAALNEINQLVNQ---           [1]
DPS3                ALW-------KDILKN-------AGKAALNEINQIVQ----           [1]
                     **           *         **** *
```

**Figure S2.** Alignment of amino acid sequences of DPS3 and other dermaseptins. The corresponding references are showed behind. "a": The sequence of DPS1 is obtained from GenBank database under the accession no. of SIW62024. The identical amino acid residues are indicated by asterisks..
